# Exploring the Trans-Cleavage Activity with Rolling Circle Amplification for Fast Detection of miRNA

**DOI:** 10.34133/bdr.0010

**Published:** 2023-03-27

**Authors:** Chenqi Niu, Juewen Liu, Xinhui Xing, Chong Zhang

**Affiliations:** ^1^ MOE Key Laboratory for Industrial Biocatalysis, Institute of Biochemical Engineering, Department of Chemical Engineering, Tsinghua University, Beijing 100084, China.; ^2^ Department of Chemistry, Waterloo Institute for Nanotechnology, University of Waterloo, 200 University Avenue West, Waterloo, ON N2L 3G1, Canada.; ^3^ Center for Synthetic and Systems Biology, Tsinghua University, Beijing 100084, China.

## Abstract

MicroRNAs (miRNAs) are a class of endogenous short noncoding RNA. They regulate gene expression and function, essential to biological processes. It is necessary to develop an efficient detection method to determine these valuable biomarkers for the diagnosis of cancers. In this paper, we proposed a general and rapid method for sensitive and quantitative detection of miRNA by combining CRISPR–Cas12a and rolling circle amplification (RCA) with the precircularized probe. Eventually, the detection of miRNA-21 could be completed in 70 min with a limit of detection of 8.1 pM with high specificity. The reaction time was reduced by almost 4 h from more than 5 h to 70 min, which makes detection more efficient. This design improves the efficiency of CRISPR–Cas and RCA-based sensing strategy and shows great potential in lab-based detection and point-of-care test.

## Introduction

MicroRNAs (miRNAs) are a class of endogenous short noncoding RNA. They regulate gene expression and function, essential to biological processes [1]. Aberrant miRNA expression has been reported in all types of tumor-associated diseases [2], indicating that miRNAs have the potential to be valuable biomarkers for the diagnosis of cancers. However, due to their sequence similarity, small size, and degradability, rapid and sensitive quantification of miRNAs is difficult. In recent years, clustered regularly interspaced short palindromic repeats (CRISPR) and CRISPR-associated proteins (Cas) have been widely used as efficient tools in nucleic acid-related detection, especially in the determination of viral infections [3]. Several nucleic acid amplification methods were generally combined with the CRISPR–Cas system to obtain higher sensitivity, such as polymerase chain reaction [4], recombinase polymerase amplification [5] and loop-mediated isothermal amplification [6,7]. In these methods, at least 1 pair of primers were designed for target detection. However, miRNAs are short single-stranded RNAs with a length of 18 to 25 nt, disallowing these methods to be directly applied for their detection.

To overcome this limitation, several miRNA detection methods based on short nucleic acid amplification techniques have been developed. For example, a method based on rolling circle transcription-Cas12a was reported by Wang and coworkers [8]. A method based on cascade amplification with a DNAzyme, an RNA ligase, an RNA polymerase, and Cas12a was reported by Sun and coworkers [9]. However, the amplification products of these 2 methods are RNAs, which are less stable and more susceptible to hydrolysis than DNA. In addition, several nucleic acid amplification methods with double-stranded DNA (dsDNA) products as an activator chain for Cas12a were reported [10–12]. Nevertheless, the protospacer adjacent motif (PAM) sequence requires to be considered when dsDNA hybridizes to CRISPR RNA–Cas12a (crRNA–Cas12a) complex, leading to complex oligonucleotide design. Although the targeting range of CRISPR–Cas12a can be improved by adapting to alternative the PAM sequences, the cleavage efficiency rate is lowered [13]. As a comparison, a single-stranded DNA (ssDNA) activator can hybridize to a crRNA–Cas12a complex even without a PAM sequence.

Rolling circle amplification (RCA) is generally recognized as an efficient isothermal amplification technique, amplifying a short DNA or RNA to form massive ssDNA using a DNA polymerase [14,15]. It is generally used for the detection of short single-stranded nucleic acids such as miRNAs [16–20]. Although it is a robust amplification method, its application is limited due to its detection time usually exceeding 5 h [8,21–23].

As an effective nuclease, the CRISPR–Cas system is known to recognize and hybridize to target nucleic acids with high specificity. Unlike Cas13a, Cas12a's target and probe are both DNA, which increases the detection assay's robustness and lowers the cost greatly. Therefore, using Cas12a to detect miRNA would be more trustworthy. In the case of the product of isothermal amplification is designed as the activator of Cas12a trans-cleavage activity, nonspecific amplification product cannot active the Cas12a fluorescence reporter system due to the specifically hybridization of Cas12a–crRNA complex to trigger the chain. In addition, Cas12a is an extremely efficient nuclease, ensuring a short reaction time. Therefore, combining RCA isothermal amplification techniques with CRISPR–Cas system would promote the performance of sensitivity, specificity, and reaction time and make it possible to develop highly sensitive, specific, and efficient biosensors for the detection of miRNAs. Recently, several CRISPR-assisted strategies for the detection of miRNA have been proposed based on rolling circle transcription and RCA [8,24,25]. Even though a favorable detection performance is obtained, the detection time (several hours) of those strategies is too long to meet the need for point-of-care testing, which is precisely the most widely used field of isothermal amplification.

**Fig. 1.**
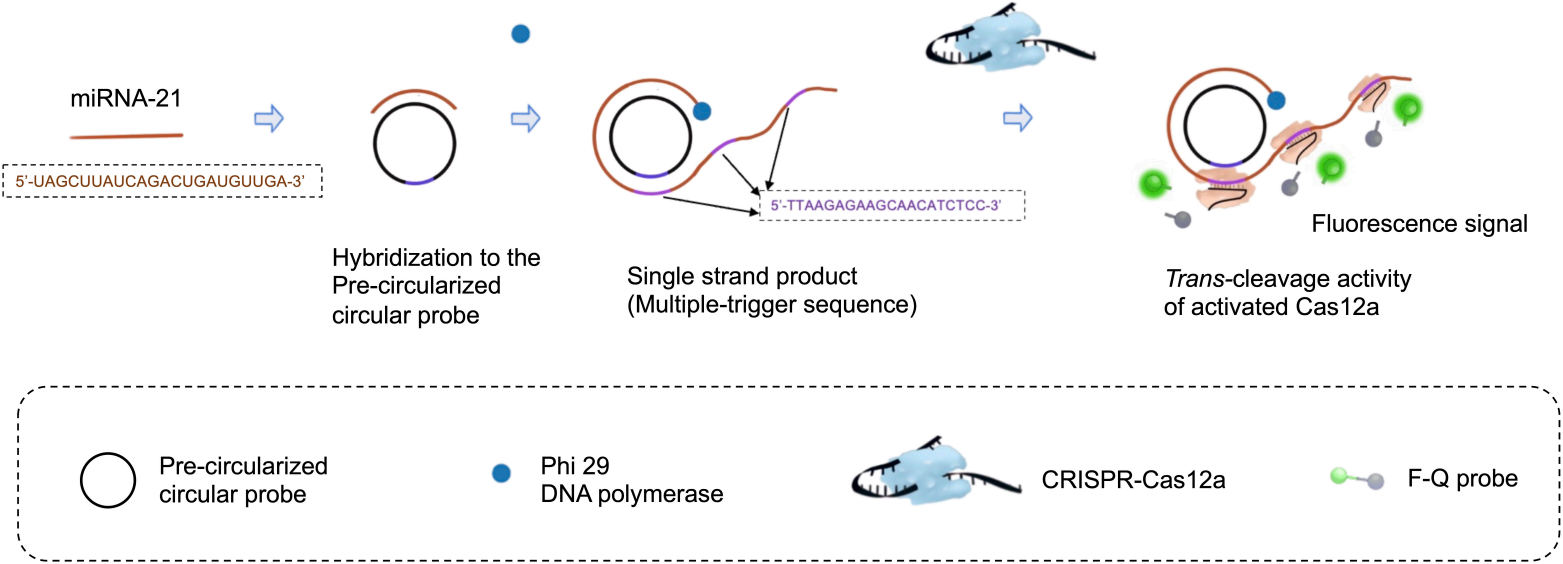
Schematic of the rRCA–Cas reaction. After hybridizing to the precircularized circular probe, the target miRNA is amplified into a long ssDNA product by RCA with massive trigger sequences repeats. The trigger sequence is designed as the activator of Cas12a for showing trans-cleavage activity. Then, the trigger chain is recognized by Cas12a–crRNA duplex and hybridizes to it. Cas12a was then triggered to demonstrate trans-cleavage activity, F-Q probe (an ssDNA probe labeled with a fluorophore at the 5′ end and a quencher at the 3′ end) was cleaved, resulting in separation of the quencher from the fluorophore, generating the fluorescence signal.

In this work, we aimed to develop a general and rapid method for sensitive quantitative detection of short-stranded nucleic acids. We combined the CRISPR–Cas12a fluorescence reporting system with RCA isothermal amplification with a precircularized probe (Fig. 1) and named it rapid RCA–Cas reaction (rRCA–Cas). As a result, the detection of miRNA could be achieved by monitoring the fluorescence signal with the limit of detection (LOD) of 8.1 pM. In addition, the reaction time was reduced by almost 4 h from more than 5 h to 70 min.

## Materials and Methods

### Reagents and materials

The F-Q probe labeled with the fluorophore and quencher was purchased from Bio-lifesci (Guangzhou, China). SYBR Green I (SGI) was purchased from Thermo Fisher Scientific (Waltham, USA). NEB CutSmart™ buffer, LbCas12a (Cas12a), Exonuclease I (EXO I), Exonuclease III (EXO III), Deoxynucleotide Triphosphates (dNTPs), T4 DNA ligase, and phi29 DNA polymerase were purchased from New England Biolabs (NEB) (USA). CircLigase was purchased from Epicentre (USA). The miRNAs, crRNAs, and oligonucleotides were synthesized from Genewiz (Suzhou, China) (Table S1). Human serum in complex matrix test was purchased from Solarbio (Beijing, China).

### Fluorescence sensitivity assay of Cas12a fluorescence reporter system compared with SYBR Green dye assay and fluorophore-labeled assay

Cas12a fluorescence reporter system: Synthetic trigger chain was diluted into different concentrations from 0 to 1 μΜ. After a 10-min preincubation of Cas12a and crRNA at room temperature, the 100-μl trans-cleavage reaction started by mixing all components, including 1× NEB CutSmart™ buffer (50 mM KAc, 20 mM tris-HCl, 10 mM MgCl_2_, and 100 μg/ml Bovine serum albumin (BSA) [pH 7.9 at 25 °C]), 30 nM LbCas12a–crRNA complex, 200 nM F-Q probe, and various concentrations of trigger chain. The fluorescence intensity of the reaction was monitored by a fluorescence plate reader for 15 min at 37 °C.

SYBR Green dye assay: The 1× SGI dye was added to a total of 100 μl of reaction with different concentrations of synthetic trigger chain from 0 to 1 μΜ. The dye binds to DNA very rapidly, reaching equilibrium within 10 min, leading to a stable fluorescence intensity. After a 10-min incubation, the fluorescence intensity was measured.

Fluorophore-labeled assay: Synthetic FAM-labeled trigger chain was diluted into different concentrations from 0 to 1 μΜ. The 100-μl solution was seated for 10 min at room temperature before the fluorescence intensity was measured.

The excitation wavelength and emission wavelength of all the fluorescent assays above were set at 492 and 518 nm, respectively.

### Precircularization of the padlock for RCA reaction

Two precircularization methods were evaluated in this study. One precircularization method was based on T4 ligase. In detail, 20 μM miRNA-21-Padlock-T4 and 20 μM RCA–helper were annealed firstly by heating at 95 °C for 5 min in 1× T4 DNA ligase buffer (50 mM tris-HCl, 10 mM MgCl_2_, 1 mM adenosine triphosphate, and 10 mM dithiothreitol (DTT) [pH 7.5 at 25 °C]) and slowly cooled down to the room temperature. Then, 10 U/μl T4 DNA ligase with 1× T4 DNA ligase buffer was added. The phosphodiester bond was formed by a 1-h incubation at 30 °C. Then, 20 U/μl EXO I with 1× Exonuclease I Reaction Buffer (67 mM Glycine-KOH, 6.7 mM MgCl_2_, and 10 mM Î^2^-ME [pH 9.5 at 25 °C]) and 50 U/μl EXO III with 1× NEBuffer™ 1 (10 mM Bis-tris-Propane-HCl, 10 mM MgCl_2_, 1 mM DTT, (pH 7 at 25 °C) were added in the mixture. After a 2-h incubation at 37 °C, the uncircularized ssDNA was degraded to purify the product. After heating at 80 °C for 20 min to inactivate the enzymes, the precircularized circular probe was stored at 4 °C until use.

The other precircularization method was based on CircLigase. The 20 μl of reaction was carried out according to the instructions. The 0.5 pmol/μl miRNA-21-Padlock-CircLigase, 50 μM adenosine triphosphate, 2.5 mM MnCl_2_, 5 U/μl CircLigase ssDNA Ligase, and 1× CircLigase reaction buffer (50 mM Mops [pH 7.5], 10 mM KCl, 5 mM MgCl_2_, and 1 mM DTT) were mixed and incubated for 1 h at 60 °C. After heating at 80 °C for 10 min to inactivate the enzyme, the precircularized circular probe was stored at 4 °C until use.

### Typical rRCA–Cas reaction

The 10-μl typical RCA reaction consisted of various concentrations of miRNA target, 50 nM circular probe with the T4 DNA ligase method, 0.25 U/μl phi29 DNA polymerase, 1× phi29 DNA Polymerase Reaction Buffer (50 mM tris-HCl, 10 mM MgCl_2_, 10 mM (NH_4_)_2_SO_4_, and 4 mM DTT [pH 7.5 at 25 °C]), and 500 μM dNTPs. After mixing evenly, the reaction was incubated for 1 h at 37 °C to obtain the amplification products.

Then, the 10-μl amplification product was incubated with a Cas12a fluorescence reporter system consisting of 30 nM Cas12a–crRNA complex, 1× NEB CutSmart™ buffer, and 200 nM F-Q probe. The mixture was incubated at 37 °C for 10 min to activate the trans-cleavage activity of Cas12a. Then, the fluorescence intensity was measured at 492-nm excitation wavelength and 518-nm emission wavelength.

### Data processing

In the experiments for optimization of the reaction parameters, *F*/*F*_0_ represents the signal-to-noise ratio, where *F* and *F*_0_ are the fluorescence intensity in the presence and absence of miRNA target, respectively.

## Results and Discussion

### Advantage of Cas12a fluorescence reporter system compared with SYBR Green dye assay and fluorophore-labeled assay

**Fig. 2.**
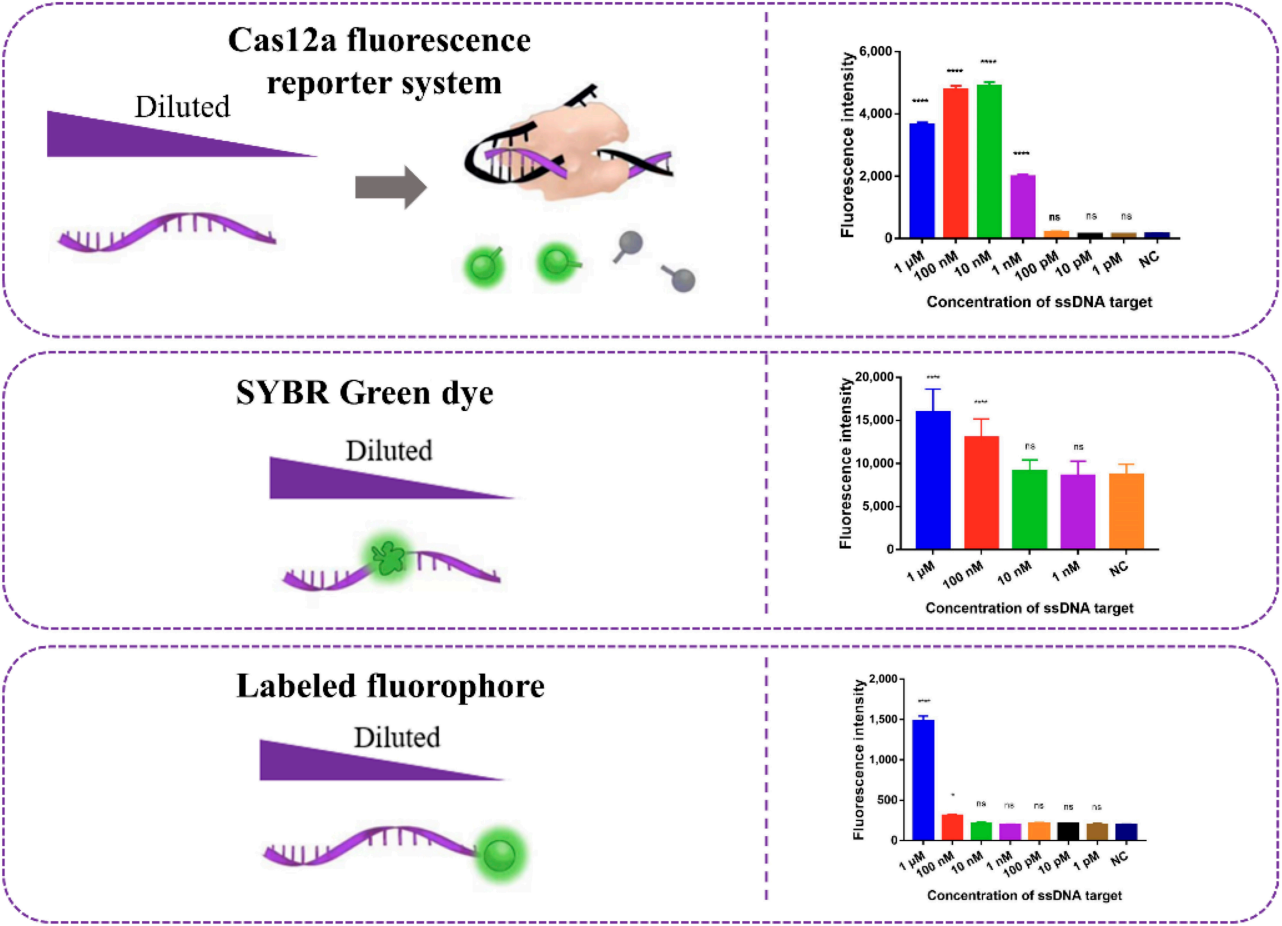
Estimation of the sensitivity of Cas12a fluorescence reporter system, SYBR Green, and FAM fluorophore with the same diluted target ssDNA. Sensitivity with Cas12a fluorescence reporter system, 1× SGI, and labeled fluorophore were tested. Error bars present the SD with 3 individual tests. NC, background signal with distilled water as a negative control. ****: *P* < 0.0001. ns, no significant difference.

SGI dye and related fluorophores were widely used in the molecule diagnosis [26–30]. We first estimated the sensitivity of the Cas12a fluorescence reporter system, the SGI dye assay, and the FAM fluorophore-labeled assay with the same series-diluted target ssDNA. As shown in Fig. 2, 100 nM target could be detected with 1× SGI and the labeled FAM fluorophore; in addition, 1 nM target could be detected with the Cas12a fluorescence reporter system. Thus, the Cas12a fluorescence reporter system greatly improved the detection sensitivity for ssDNA target detection. The results indicated the Cas12a fluorescence reporter system has the potential to be used to develop highly sensitive biosensors. In addition, the fluorescence signal from the Cas12a fluorescence reporter system did not increase monotonically with the increase of target concentration. The activity of Cas12a was inhibited at a high concentration of DNA target, which was consistent with our previous report [31].

### Schematic and feasibility of rRCA–Cas reaction

**Fig. 3.**
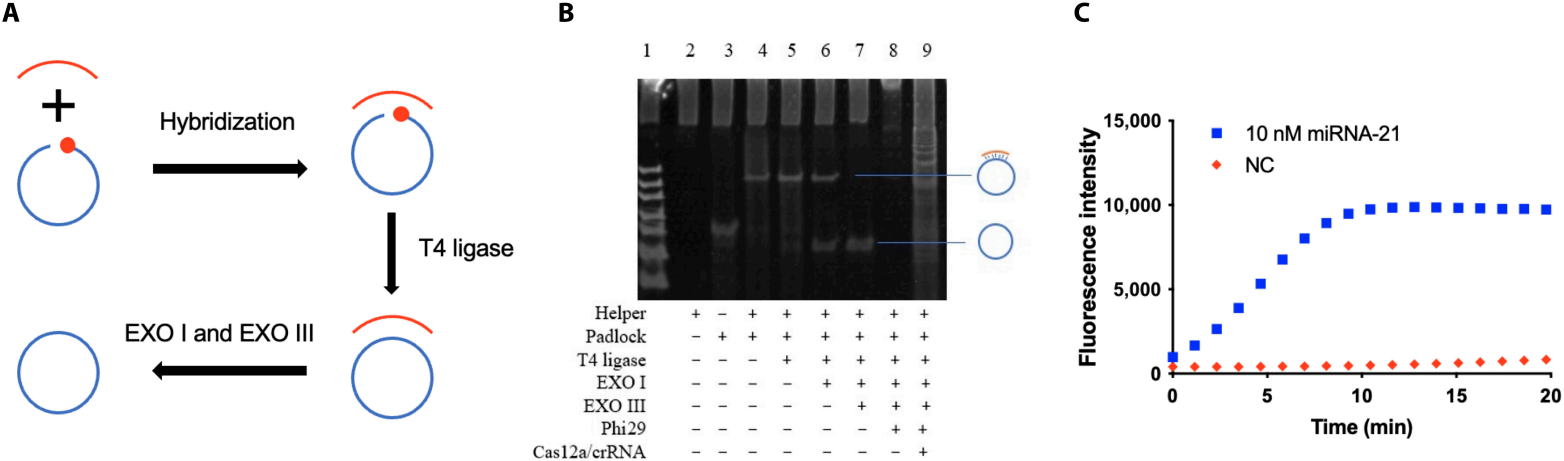
Feasibility of rRCA–Cas reaction. (A) The workflow of precircularization of the padlock to obtain circular probe. (B) Verification by the 12% native PAGE image. Lane 1: ssDNA ladder. Lane 2: 1 μM helper chain. Lane 3: 1 μM linear padlock chain. Lane 4: After incubation of helper and padlock chain. Lane 5: Padlock was circularized to circular probe by T4 DNA ligase with helper hybridized on it. Lanes 6 and 7: After EXO I and EXO III hydrolysis, circular probe without helper on it was observed. Lane 8: The RCA product of 10 nM target miRNA-21. Lane 9: The RCA product was cleaved by Cas12a/crRNA complex. (C) Verification by the fluorescence spectrum. The blue trace represents the signal of the entire rRCA–Cas reaction in the presence of the target, which is the same as lane 9 in the PAGE image. The orange trace represents the signal in the absence of the target. NC indicates the blank signal without the target in the reaction.

In the traditional RCA approach, the padlock probe was designed to hybridize with miRNA target at both the 3′ and 5′ ends [21,32–34]. The following steps including ligation to form a circular DNA template and hydrolyze excess free DNA are time-consuming leading to a total detection time of about 5 h [21,22]. Herein, we employed a precircularization step to save detection time (Fig. 3A). The padlock probe was designed to hybridize with a helper DNA chain and ligated by T4 DNA ligase. EXO I is often used to hydrolyze ssDNA, and EXO III is used to hydrolyze nonspecific complexes [35–37]. After hydrolysis of excess free DNA by EXO I and EXO III, a precircularized circular probe acting as a rolling template was obtained. In addition, the activated Cas12a can cleavage very fast as an efficient nuclease. Combining these 2 advantages, this proposed approach can decrease the detection time to about 70 min.

The schematic of rRCA–Cas strategy is shown in Fig. 1. The circular probe DNA was precircularized to shorten the detection time. In the presence of target miRNA (dark orange line), after hybridizing to the precircularization circular probe functional as the template, miRNA was extended by phi29 DNA polymerase to initiate the RCA. A long ssDNA sequence was generated, containing numerous “trigger” (dark purple line) repeats. Then, the repeat trigger sequences hybridized to the Cas12a–crRNA by base complementation. Then, Cas12a showed trans-cleavage activity, cleaving the ssDNA around it, including the F-Q probe. As a result, the quencher was separated from the fluorophore, increasing the fluorescence signal.

The miRNA-21 was used to demonstrate the feasibility of the rRCA–Cas reaction for miRNA detection, and the feasibility was first verified via 12% native polyacrylamide gel electrophoresis (PAGE) (Fig. 3B). The helper–padlock complex was formed and showed a band higher and a single padlock chain in lane 4. Ligation of padlock did not change the molecular weight of the complex as well as the band in lane 5. EXO I enzyme catalyzes and removes nucleotides in the 3′ to 5′ direction from linear ssDNA, and EXO III catalyzes and removes nucleotides in the 3′ to 5′ direction from linear or nicked dsDNA. After hydrolysis of EXO I and EXO III, a circular probe was obtained in lane 7. For the miRNA-21 detection, 10 nM miRNA target and phi29 DNA polymerase were added, and a long ssDNA product was observed in lane 8. Then, the Cas12a fluorescence reporter system was added, and the long ssDNA amplification product was cleaved by Cas12a, which led to a few kinds of fragments with different molecular weights in lane 9. Then, we employed the fluorescence spectrum to verify the feasibility (Fig. 3C). The fluorescence signal was obtained in the presence of miRNA target (blue trace). However, in the absence of miRNA target, the RCA was not initiated, resulting in no generation of the long ssDNA. The Cas12a was inactive and no fluorescence signal increased (red trace). In addition, another circularization method was investigated in which the padlock was directly circularized by the CircLigase enzyme without the helper chain (Fig. S1). It proved to be as effective as the T4 DNA ligase-based method described above. In consideration of cost, we chose T4 DNA ligase-based method for further study.

### Optimization of rRCA–Cas reaction

**Fig. 4.**
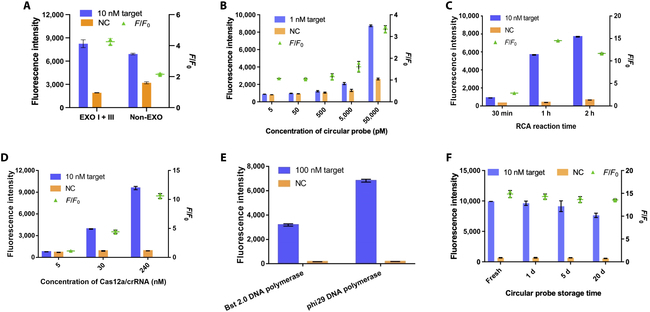
Optimization of rRCA–Cas reaction. (A) Evaluation of the impact of EXO I and III on rRCA–Cas reaction. (B) The fluorescence intensity and *F*/*F*_0_ values of various circular probe concentrations. (C) The fluorescence intensity and *F*/*F*_0_ values of various amplification times of RCA reaction. (D) The fluorescence intensity and *F*/*F*_0_ values of various Cas12a–crRNA complex concentrations. (E) The performances with different DNA polymerases used in the rRCA–Cas reaction. (F) The fluorescence intensity and *F*/*F*_0_ values of various storage times of circular probe. NC indicates the blank signal without the target in the reaction. *F*/*F*_0_ means the fluorescence intensity ratio of the group with miRNA target and the group without miRNA target (NC), which represents the signal-to-noise ratio. Error bars present the SD with 3 individual tests.

We further optimized the reaction system to achieve the best sensitivity performance. Firstly, the impact of EXO I and EXO III was investigated (Fig. 4A). EXO enzymes were essential to decrease background signal intensity by hydrolyzing free DNAs and the helper chain on the circular probe. Secondly, we estimated the effect of circular probe concentration (Fig. 4B). The results showed that the fluorescence intensity increased as the concentration of the circular probe increased, and 50 nM circular probe was selected for further experiment. Then, the reaction time of RCA was evaluated, and the results indicated that 1 h was optimal by *F*/*F*_0_ value (Fig. 4C). The concentration of Cas12a–crRNA complex in the Cas12a fluorescence reporter system was also evaluated (Fig. 4D). The fluorescence intensity increased as the concentration of the Cas12a–crRNA increased, and 240 nM Cas12a–crRNA complex had the best *F*/*F*_0_ value. However, 30 nM complex was used for further experiment for cost consideration. Moreover, we used Bst 2.0 DNA polymerase to replace phi29 DNA polymerase, so that the RCA reaction could be carried out at 65 °C instead of 37 °C (Fig. 4E). However, the amplification efficiency of Bst 2.0 DNA polymerase was lower than that of phi29 DNA polymerase in our assay. In addition, RCA at 37 °C is consistent with the reaction temperature of Cas12a. Thus, phi29 DNA polymerase was used for further study. We further investigate the impact of the storage time on the stability of the circular probe (Fig. 4F). The fluorescence intensity kept stable within 5 d. After 20 d of storage at 4 °C, there is a modest drop in fluorescence intensity, indicating that the circular probe has degraded slightly. Under the optimal conditions determined above, the whole assay was performed at 37 °C and the detection time was 70 min (1 h for RCA and 10 min for the Cas12a fluorescence reporter system).

### Sensitivity, specificity, and real-sample test of rRCA–Cas reaction

**Fig. 5.**
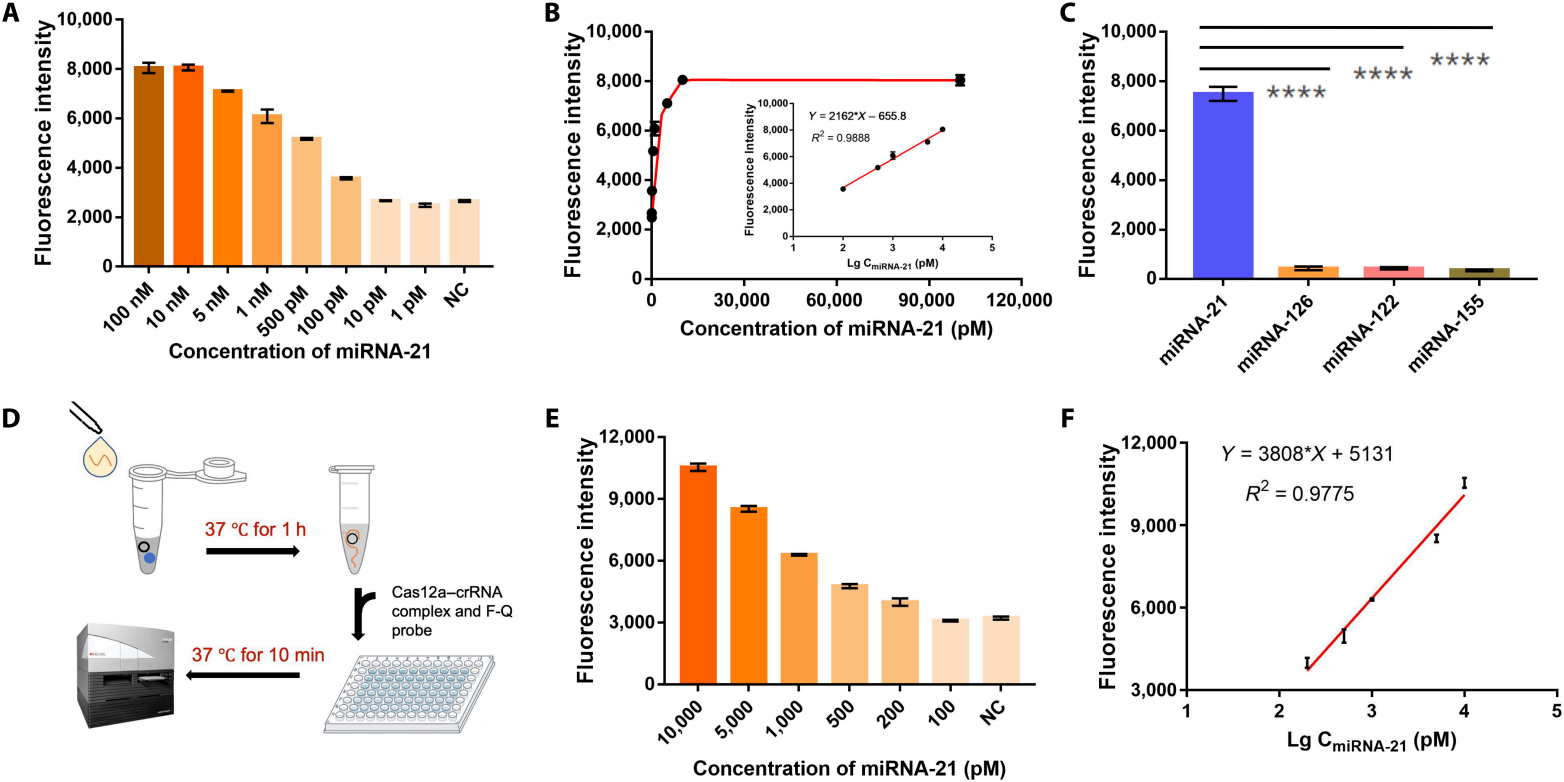
The performance of the rRCA–Cas reaction for detecting miRNA-21. (A) Fluorescence intensity at varied target miRNA concentrations ranging from 1 pM to 100 nM under buffer conditions. (B) The calibration curve of fluorescence intensity and varied target miRNA concentrations. Inset: Linear range from 100 pM to 10 nM, *R*^2^ = 0.9888. (C) Specificity evaluation for 5 nM miRNAs detection with different miRNA species (miRNA-21, miRNA-126, miRNA-155, and miRNA-122). ****: *P* < 0.0001. (D) Workflow for miRNA detection in serum. (E) Fluorescence intensity at varied target miRNA concentrations ranging from100 pM to 10 nM in 1% human serum. (F) The linear range from 200 pM to 10 nM regressed from the data in (E), *R*^2^ = 0.9775. Error bars present the SD with 3 individual tests. NC indicates the blank signal without the target in the reaction.

Different concentrations of synthesized target miRNAs (100 nM, 10 nM, 5 nM, 1 nM, 500 pM, 100 pM, 10 pM, and 1 pM) were used to evaluate the sensitivity (Fig. 5A). The fluorescence intensity was measured for 15 min for quantitative analysis following the addition of the Cas12a fluorescent system. Fluorescence intensity was plotted against miRNA-21 concentrations, and the regression of the best linear fit was between 100 pM and 10 nM (Fig. 5B). Based on a 3*SD/slope, the LOD was determined to be 8.1 pM. Although this method could not achieve a higher sensitivity than some reported nanomaterials-based and electrochemistry-based ones for the detection of miRNAs [38–40], the LOD as low as 8.1 pM of this method is comparable to fluorescence detection comparing to previous work [8,22,24,41–44]. Most importantly, our detection time is less than the vast majority of reported biosensors based on RCA (see Table S2 for details).

To investigate the specificity of the rRCA–Cas reaction, a variety of miRNAs including miRNA-21, miRNA-126, miRNA-122, and miRNA-155 were tested, which have the potential to be the biomarkers of colorectal cancer, venous thromboembolism of persistent, liver diseases, and B-cell malignancies. The concentrations of these miRNAs were set to be the same at 5 nM to compare the fluorescence signal intensity. As seen in Fig. 5C, miRNA-21 had a fluorescence signal that was 19.3-fold (*P* < 0.0001) than miRNA-126, miRNA-122, and miRNA-155, showing this method successfully achieved high specificity for miRNA-21. Given the high specificity of the DNA ligase, the high specificity exhibited by the reaction was expected [45,46].

The fluorescence signal of miRNA-21 was 19.3-fold higher (*P* < 0.0001), while no fluorescence signals were detected for miRNA-126, miRNA-122, and miRNA-155, indicating that high selectivity of miRNA-21 was achieved for this strategy. Both of the 2 padlock precircularization methods—T4 DNA ligase-based and CircLigase-based—showed high specificity (Fig. S1D).

To verify the feasibility and investigate the matrix effect of rRCA–Cas reaction in real samples, we detected different concentration of miRNAs in 1% human serum. The workflow is simple and the miRNA can be detected in 70 min (Fig. 5D). Fluorescence intensity was plotted against miRNA-21 concentrations, and the regression of the best linear fit was between 200 pM and 10 nM (Fig. 5, E and F). The recovery of the strategy was investigated by spiking various concentrations of miRNA-21 in 1% human serum. The estimated recoveries ranged from 89.5% to 102.3% (Table S3).

## Conclusion

In this study, we proposed a detection strategy for achieving rapid determination of miRNA via the CRISPR–Cas12a fluorescence reporting system coupled with a precircularized probe in RCA. Finally, with a LOD of 8.1 pM and high specificity, miRNA-21 was quantitatively identified in 70 min at 37 °C. The reaction time was reduced by almost 4 h from more than 5 h to 70 min by precircularizing the padlock to obtain the circular probe, which makes detection more efficient. Moreover, we fully illustrated the advantages of using Cas12a as an additional module for isothermal amplification, besides the improved sensitivity compared to the SYBR Green and fluorophore. In addition, the trigger sequence can be designed as the same sequence in our proposed approach, and the identification of different miRNAs can be achieved by simply replacing the padlock chain. The Cas12a fluorescence reporting system could be used as a general additional module without any change, which made our approach more convenient and time-saving.

In addition, we have reported an aptamer-based method for the detection of β-estrodial [47], which was based on the aptamer structure-switch binding to β-estrodial. It can serve as a recognition element for non-nucleic acid molecules to develop the biosensor. Furthermore, the aptamer-based structure-switch recognition element could be integrated with the proposed rRCA–Cas reaction, developing of non-nucleic acid molecule biosensors besides the detection of miRNAs.

## References

[1] Ahmed FE. Role of miRNA in carcinogenesis and biomarker selection: A methodological view. Expert Rev Mol Diagn. 2007;7(5):569–603. 17892365 10.1586/14737159.7.5.569

[2] Cheng L, Sharples RA, Scicluna BJ, Hill AF. Exosomes provide a protective and enriched source of miRNA for biomarker profiling compared to intracellular and cell-free blood. J Extracell Vesicles. 2014;3(1):23743. 10.3402/jev.v3.23743 PMC3968297 24683445

[3] Dronina J, Samukaite-Bubniene U, Ramanavicius A. Advances and insights in the diagnosis of viral infections. J Nanobiotechnol. 2021;19(1):348. 10.1186/s12951-021-01081-2 PMC8556785 34717656

[4] Chen JS, Ma E, Harrington LB, Da Costa M, Tian X, Palefsky JM, Doudna JA. CRISPR-Cas12a target binding unleashes indiscriminate single-stranded DNase activity. Science. 2018;360(6387):436. 29449511 10.1126/science.aar6245 PMC6628903

[5] Gootenberg JS, Abudayyeh OO, Lee JW, Essletzbichler P, Dy AJ, Joung J, Verdine V, Donghia N, Daringer NM, Freije CA, et al. Nucleic acid detection with CRISPR-Cas13a/C2c2. Science. 2017;356(6336):438. 28408723 10.1126/science.aam9321 PMC5526198

[6] Qian C, Wang R, Wu H, Zhang F, Wu J, Wang L. Uracil-mediated new photospacer-adjacent motif of Cas12a to realize visualized DNA detection at the single-copy level free from contamination. Anal Chem. 2019;91(17):11362–11366. 31403279 10.1021/acs.analchem.9b02554

[7] Mukama O, Wu J, Li Z, Liang Q, Yi Z, Lu X, Liu Y, Liu Y, Hussain M, Makafe GG, et al. An ultrasensitive and specific point-of-care CRISPR/Cas12 based lateral flow biosensor for the rapid detection of nucleic acids. Biosens Bioelectron. 2020;159:112143. 32364943 10.1016/j.bios.2020.112143

[8] Wang G, Tian W, Liu X, Ren W, Liu C. New CRISPR-derived microRNA sensing mechanism based on Cas12a self-powered and rolling circle transcription-unleashed real-time crRNA recruiting. Anal Chem. 2020;92(9):6702–6708. 32272843 10.1021/acs.analchem.0c00680

[9] Sun H-H, He F, Wang T, Yin B-C, Ye B-C. A Cas12a-mediated cascade amplification method for microRNA detection. Analyst. 2020;145(16):5547–5552. 32609115 10.1039/d0an00370k

[10] Chen M, Luo R, Li S, Li H, Qin Y, Zhou D, Liu H, Gong X, Chang J. Paper-based strip for ultrasensitive detection of OSCC-associated salivary microRNA via CRISPR/Cas12a coupling with IS-primer amplification reaction. Anal Chem. 2020;92(19):13336–13342. 32809800 10.1021/acs.analchem.0c02642

[11] Zhang M, Wang H, Wang H, Wang F, Li Z. CRISPR/Cas12a-assisted ligation-initiated loop-mediated isothermal amplification (CAL-LAMP) for highly specific detection of microRNAs. Anal Chem. 2021;93(22):7942–7948. 34038095 10.1021/acs.analchem.1c00686

[12] Peng S, Tan Z, Chen S, Lei C, Nie Z. Integrating CRISPR-Cas12a with a DNA circuit as a generic sensing platform for amplified detection of microRNA. Chem Sci. 2020;11(28):7362–7368. 33133487 10.1039/d0sc03084h PMC7553042

[13] Dronina J, Samukaite-Bubniene U, Ramanavicius A. Towards application of CRISPR-Cas12a in the design of modern viral DNA detection tools (review). J Nanobiotechnol. 2022;20(1):41. 10.1186/s12951-022-01246-7 PMC8777428 35062978

[14] Van Ness J, Van Ness Lori K, Galas David J. Isothermal reactions for the amplification of oligonucleotides. Proc Natl Acad Sci U S A. 2003;100(8):4504–4509. 12679520 10.1073/pnas.0730811100 PMC404692

[15] Dronina J, Bubniene US, Ramanavicius A. The application of DNA polymerases and Cas9 as representative of DNA-modifying enzymes group in DNA sensor design (review). Biosens Bioelectron. 2021;175:112867. 33303323 10.1016/j.bios.2020.112867

[16] Deng R, Zhang K, Li J. Isothermal amplification for MicroRNA detection: From the test tube to the cell. Acc Chem Res. 2017;50(4):1059–1068. 28355077 10.1021/acs.accounts.7b00040

[17] Wei S, Chen G, Jia X, Mao X, Chen T, Mao D, Zhang W, Xiong W. Exponential amplification reaction and triplex DNA mediated aggregation of gold nanoparticles for sensitive colorimetric detection of microRNA. Anal Chim Acta. 2020;1095:179–184. 31864620 10.1016/j.aca.2019.10.020

[18] Zhang Y, Zhang C-y. Sensitive detection of microRNA with isothermal amplification and a single-quantum-dot-based nanosensor. Anal Chem. 2012;84(1):224–231. 22103863 10.1021/ac202405q

[19] Zhang P, Wu X, Yuan R, Chai Y. An “off–on” electrochemiluminescent biosensor based on DNAzyme-assisted target recycling and rolling circle amplifications for ultrasensitive detection of microRNA. Anal Chem. 2015;87(6):3202–3207. 25679541 10.1021/ac504455z

[20] Xu H, Wu D, Zhang Y, Shi H, Ouyang C, Li F, Jia L, Yu S, Wu Z-S. RCA-enhanced multifunctional molecule beacon-based strand-displacement amplification for sensitive microRNA detection. Sensors Actuators B Chem. 2018;258:470–477.

[21] Li X, Wang L, Li C. Rolling-circle amplification detection of thrombin using surface-enhanced Raman spectroscopy with core-shell nanoparticle probe. Chem Eur J. 2015;21(18):6817–6822. 25766032 10.1002/chem.201405884

[22] Song H, Zhang Y, Wang S, Huang K, Luo Y, Zhang W, Xu W. Label-free polygonal-plate fluorescent-hydrogel biosensor for ultrasensitive microRNA detection. Sensors Actuators B Chem. 2020;306:127554.

[23] Ciftci S, Cánovas R, Neumann F, Paulraj T, Nilsson M, Crespo GA, Madaboosi N. The sweet detection of rolling circle amplification: Glucose-based electrochemical genosensor for the detection of viral nucleic acid. Biosens Bioelectron. 2020;151:112002. 31999596 10.1016/j.bios.2019.112002

[24] Jiang W, Chen Z, Lu J, Ren X, Ma Y. Ultrasensitive visual detection of miRNA-143 using a CRISPR/Cas12a-based platform coupled with hyperbranched rolling circle amplification. Talanta. 2023;251:123784. 35988346 10.1016/j.talanta.2022.123784

[25] Zhang G, Zhang L, Tong J, Zhao X, Ren J. CRISPR-Cas12a enhanced rolling circle amplification method for ultrasensitive miRNA detection. Microchem J. 2020;158:105239.

[26] Zhang C, Zhang H, Wu P, Zhang X, Liu J. Suppressing the background activity of hemin for boosting the sensitivity of DNAzyme-based biosensors by SYBR Green I. Biosens Bioelectron. 2020;169:112603. 32947082 10.1016/j.bios.2020.112603

[27] Kong L, Xu J, Xu Y, Xiang Y, Yuan R, Chai Y. A universal and label-free aptasensor for fluorescent detection of ATP and thrombin based on SYBR Green Idye. Biosens Bioelectron. 2013;42:193–197. 23202351 10.1016/j.bios.2012.10.064

[28] Chen J, Ji X, He Z. Smart composite reagent composed of double-stranded DNA-templated copper nanoparticle and SYBR Green I for hydrogen peroxide related biosensing. Anal Chem. 2017;89(7):3988–3995. 28264556 10.1021/acs.analchem.6b04484

[29] Asnaashari M, Esmaeilzadeh Kenari R, Farahmandfar R, Taghdisi SM, Abnous K. Fluorescence quenching biosensor for acrylamide detection in food products based on double-stranded DNA and gold nanoparticles. Sensors Actuators B Chem. 2018;265:339–345.

[30] Wu Y, Chen X, Wang X, Yang M, Xu F, Hou C, Huo D. A fluorescent biosensor based on prismatic hollow metal-polydopamine frameworks and 6-carboxyfluorescein (FAM)-labeled protein aptamer for CA15-3 detection. Sensors Actuators B Chem. 2021;329:129249.

[31] Niu C, Wang C, Li F, Zheng X, Xing X, Zhang C. Aptamer assisted CRISPR-Cas12a strategy for small molecule diagnostics. Biosens Bioelectron. 2021;183:113196. 33839534 10.1016/j.bios.2021.113196

[32] Lizardi PM, Huang X, Zhu Z, Bray-Ward P, Thomas DC, Ward DC. Mutation detection and single-molecule counting using isothermal rolling-circle amplification. Nat Genet. 1998;19(3):225–232. 9662393 10.1038/898

[33] Neubacher S, Arenz C. Rolling-circle amplification: Unshared advantages in miRNA detection. Chembiochem. 2009;10(8):1289–1291. 19373796 10.1002/cbic.200900116

[34] Li X-H, Zhang X-L, Wu J, Lin N, Sun W-M, Chen M, Ou Q-S, Lin Z-Y. Hyperbranched rolling circle amplification (HRCA)-based fluorescence biosensor for ultrasensitive and specific detection of single-nucleotide polymorphism genotyping associated with the therapy of chronic hepatitis B virus infection. Talanta. 2019;191:277–282. 30262063 10.1016/j.talanta.2018.08.064

[35] Zhang J, Fan Y, Li J, Huang B, Wen H, Ren J. Cascade signal enhancement by integrating DNA walking and RCA reaction-assisted “silver-link” crossing electrode for ultrasensitive electrochemical detection of *Staphylococcus aureus*. Biosens Bioelectron. 2022;217:114716. 36126557 10.1016/j.bios.2022.114716

[36] Gao T, Chai W, Shi L, Shi H, Sheng A, Yang J, Li G. A new colorimetric assay method for the detection of anti-hepatitis C virus antibody with high sensitivity. Analyst. 2019;144(21):6365–6370. 31566645 10.1039/c9an01466g

[37] Li X-Y, Du Y-C, Zhang Y-P, Kong D-M. Dual functional Phi29 DNA polymerase-triggered exponential rolling circle amplification for sequence-specific detection of target DNA embedded in long-stranded genomic DNA. Sci Rep. 2017;7(1):6263. 28740223 10.1038/s41598-017-06594-1 PMC5524717

[38] Norouzi M, Yasamineh S, Montazeri M, Dadashpour M, Sheervalilou R, Abasi M, Pilehvar-Soltanahmadi Y. Recent advances on nanomaterials-based fluorimetric approaches for microRNAs detection. Mater Sci Eng C. 2019;104:110007. 10.1016/j.msec.2019.110007 31500008

[39] Shabaninejad Z, Yousefi F, Movahedpour A, Ghasemi Y, Dokanehiifard S, Rezaei S, Aryan R, Savardashtaki A, Mirzaei H. Electrochemical-based biosensors for microRNA detection: Nanotechnology comes into view. Anal Biochem. 2019;581:113349. 31254490 10.1016/j.ab.2019.113349

[40] Alim S, Vejayan J, Yusoff MM, Kafi AKM. Recent uses of carbon nanotubes & gold nanoparticles in electrochemistry with application in biosensing: A review. Biosens Bioelectron. 2018;121:125–136. 30205246 10.1016/j.bios.2018.08.051

[41] Ouyang W, Liu Z, Zhang G, Chen Z, Guo L, Lin Z, Qiu B, Chen G. Enzyme-free fluorescent biosensor for miRNA-21 detection based on MnO_2_ nanosheets and catalytic hairpin assembly amplification. Anal Methods. 2016;8(48):8492–8497.

[42] Liu Y, Shen T, Li J, Gong H, Chen C, Chen X, Cai C. Ratiometric fluorescence sensor for the microRNA determination by catalyzed hairpin assembly. ACS Sensors. 2017;2(10):1430–1434. 28936869 10.1021/acssensors.7b00313

[43] Cai B, Guo S, Li Y. MoS_2_-based sensor for the detection of miRNA in serum samples related to breast cancer. Anal Methods. 2018;10(2):230–236.

[44] Bahari D, Babamiri B, Salimi A, Rashidi A. Graphdiyne/graphene quantum dots for development of FRET ratiometric fluorescent assay toward sensitive detection of miRNA in human serum and bioimaging of living cancer cells. J Lumin. 2021;239:118371.

[45] Zhao J, He Y, Tan K, Yang J, Chen S, Yuan R. Novel ratiometric electrochemiluminescence biosensor based on BP-CdTe QDs with dual emission for detecting microRNA-126. Anal Chem. 2021;93(36):12400–12408. 34469691 10.1021/acs.analchem.1c02408

[46] Wei H, Tang S, Hu T, Zhao G, Guan Y. Production of dumbbell probe through hairpin cleavage-ligation and increasing RCA sensitivity and specificity by circle to circle amplification. Sci Rep. 2016;6(1):29229. 27385060 10.1038/srep29229 PMC4935871

[47] Niu C, Zhang C, Liu J. Capture-SELEX of DNA aptamers for estradiol specifically and estrogenic compounds collectively. Environ Sci Technol. 2022;56(24):17702–17711. 36441874 10.1021/acs.est.2c05808

