# Supplementary material for: Exploring the Trans-Cleavage Activity with Rolling Circle Amplification for Fast Detection of miRNA
Source: Biodes Res. 2023 Mar 27;5:0010. doi: 10.34133/bdr.0010 (PMC10085249; doi:10.34133/bdr.0010)
Supplement: Supplementary Materials — Fig. S1. Tables S1 to S3. [file bdr.0010.f1.docx]

**Supplementary Materials**

Exploring the Trans-Cleavage Activity with Rapid Rolling Circle Amplification for Sensing of miRNA

Chenqi Niu^a,c^, Juewen Liu^c^, Xinhui Xing^a, b^, Chong Zhang^a, b,^ *

^a^MOE Key Laboratory for Industrial Biocatalysis, Institute of Biochemical Engineering, Department of Chemical Engineering, Tsinghua University, Beijing, 100084, China

^b^Center for Synthetic and Systems Biology, Tsinghua University, Beijing, 100084, China

^c^Department of Chemistry, Waterloo Institute for Nanotechnology, University of Waterloo, 200 University Avenue West, Waterloo, Ontario N2L 3G1, Canada

1. **Supplementary figures**


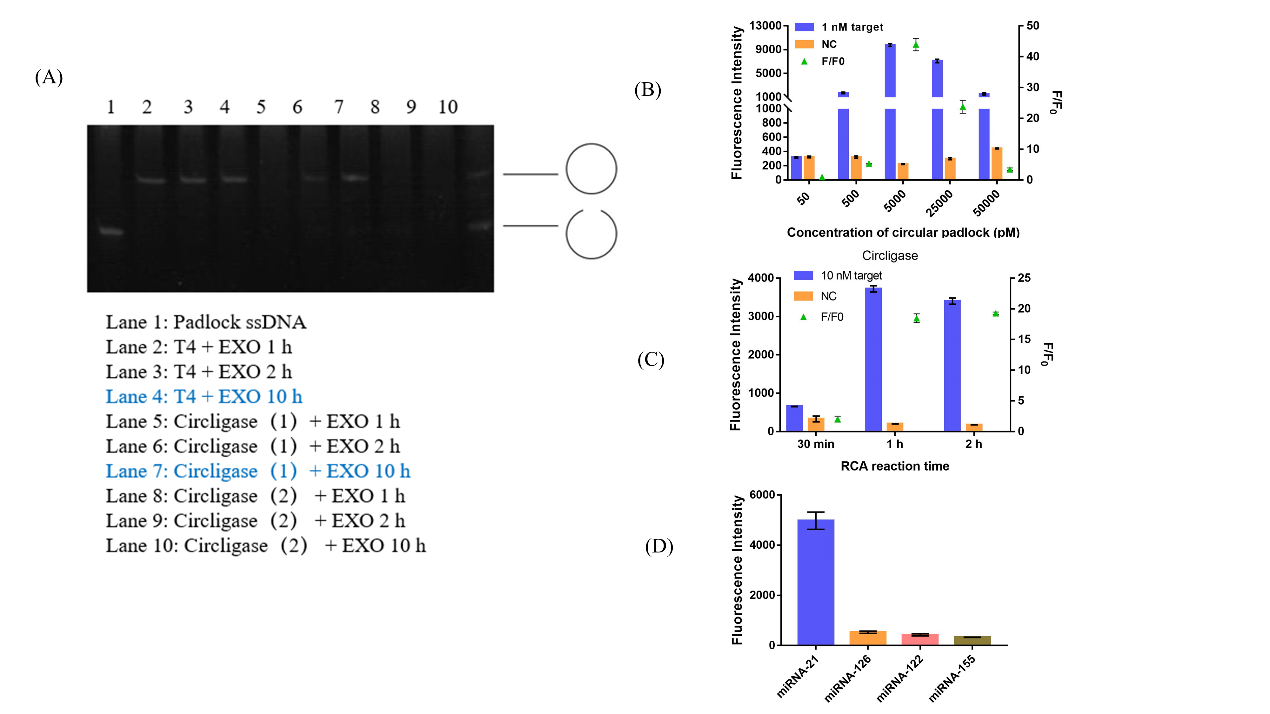


Figure S1. Evaluation of pre-circularization method based on Circligase in RCA-Cas reaction. (A) performance with different ligase and EXO incubation time. It indicated 2h EXO incubation time was enough for T4 ligase (Quantitative analysis of the gel band by ImageJ, data not shown). And Circligase (1) (purchased form Epicentre) with 10 h EXO incubation time could obtain pure circularized padlock template. Another Circligase (2) (purchased form HaiGene, for avoiding confusion, not mentioned in the manuscript) didn’t work for every conditions. (B) Optimization of padlock concentration in amplification. (C) Optimization of reaction time in amplification. (D) Specificity evaluation for 5 nM miRNAs detection with different miRNA species (miRNA-21, miRNA-126, miRNA-122 and miRNA-155). The results above showed that circligase-based method is effective as the T4 DNA ligase-based method. In consideration of cost, we chose T4 DNA ligase-based method for further study.

1. **Supplementary tables**

Table S1. The sequence of crRNAs, miRNAs and oligonucleotides

| Name | Sequence (5’-3’) |
| --- | --- |
| Trigger | TTAAGAGAAGCAACATCTCC |
| crRNA | UAAUUUCUACUAAGUGUAGAUGGAGAUGUUGCUUCUCUUAA |
| F-Q probe | FAM-TTTTTT-BHQ1 |
| Trigger-FAM | FAM- TTAAGAGAAGCAACATCTCC |
| miRNA-21 | UAGCUUAUCAGACUGAUGUUGA |
| miRNA-126 | UCGUACCGUGAGUAAUAAUGCG |
| miRNA-122 | UGGAGUGUGACAAUGGUGUUUG |
| miRNA-155 | UUAAUGCUAAUCGUGAUAGGGG |
| miRNA-21-Padlock-T4 | P-AAAAAAAAAAAAAAATCAACATCAGTCTGATAAGCTAAAAGGAGATGTTGCTTCTCTTAAAAAAAAAAAAAAAAA |
| RCA-helper | TTTTTTTTTTTTTTTTTTTT-P |
| miRNA-21-Padlock-circligase | P-CTGATAAGCTAAAAAAAAAGGAGATGTTGCTTCTCTTAAAAAAAAAATCAACATCAGT |

“P” indicated phosphorylation modification.

Table S2. Comparison of sensitivities and detection time of different detection methods for miRNAs.

| No. | Signal output | Characteristic of the method | LOD | Working range | Detection time | Reference |
| --- | --- | --- | --- | --- | --- | --- |
| 1 | Fluorescence | Structure switch with Complementary chain | nM | Not mentioned | 10 min | Larkey et al., 2014 |
| 2 | Fluorescence | HCR on MnO_2_ nanosheets | 0.33 nM | 1 to 50 nM | More than 2 h | Ouyanget al., 2016 |
| 3 | Fluorescence | catalyzed hairpin assembly-based | 72 pM | 0.5 to 50 nM | More than 1.5 h | Liu et al., 2017 |
| 4 | Fluorescence | Structure switch with RNA aptamer | nM | Not mentioned | About 45 min | Ying et al., 2017 |
| 5 | Fluorescence | Hybridize to FAM-DNA on MoS_2_ | 500 pM | 1 to 40 ng/mL | About 40 min | Cai et al., 2014 |
| 6 | Fluorescence | Photothermal immunoassay (PTIA) RCA on polygonal-plate fluorescent-hydrogel | 5 pM | 0.5 to 50 nM | More than 6 h | Song et al., 2020 |
| 7 | Fluorescence | FRET of Graphdiyne/graphene quantum dots | 0.5 pM | 5 pM to 200 nM | About 1 h | Bahari et al., 2021 |
| 8 | Fluorescence | Surface acoustic wave (SAW) based | 0.19 nM | 0.5 to 5 nM | About 1.5 h | Gamze et al., 2021 |
| 9 | Fluorescence | RCA combined with CRISPR | 34.7 fM | 10 fM ~ 1 nM | About 4.5 h | Zhang et al., 2022 |
| 10 | UV | Hyperbranched RCA combined with CRISPR | fM level by eye and aM level by UV | Not mentioned | More than 5 h | Jiang et al., 2023 |
| 11 | Fluorescence | RCT combined with CRISPR | aM level | Not mentioned | About 6 h | Wang et al., 2020 |
| 10 | Fluorescence | RCA-Cas strategy | 8.1 pM | 100 pM to 10 nM | 70 min | This work |

Table S3. Recovery experiments of miRNA 21 spiked in 1% human serum with RCA-Cas reaction.

| Matrix | Added (pM) | Found ((pM) | Recovery (%) |
| --- | --- | --- | --- |
| 1% human serum | 1000 | 1023.05±133.61 | 102.30±13.36 |
| 1% human serum | 500 | 462.16±39.36 | 92.43±7.87 |
| 1% human serum | 200 | 178.92±10.29 | 89.46±5.14 |

**Reference**

1. Larkey N E, Almlie C K, Tran V, et al. Detection of miRNA using a double-strand displacement biosensor with a self-complementary fluorescent reporter[J]. Analytical chemistry, 2014, 86(3): 1853-1863.
2. Ouyang W, Liu Z H, Zhang G F, et al. Enzyme-free fluorescent biosensor for miRNA-21 detection based on MnO 2 nanosheets and catalytic hairpin assembly amplification[J]. Analytical Methods, 2016, 8(48): 8492-8497.
3. Liu Y, Shen T, Li J, et al. Ratiometric fluorescence sensor for the microRNA determination by catalyzed hairpin assembly[J]. ACS sensors, 2017, 2(10): 1430-1434.
4. Ying Z M, Wu Z, Tu B, et al. Genetically encoded fluorescent RNA sensor for ratiometric imaging of microRNA in living tumor cells[J]. Journal of the American Chemical Society, 2017, 139(29): 9779-9782.
5. Cai B, Guo S, Li Y. MoS 2-based sensor for the detection of miRNA in serum samples related to breast cancer[J]. Analytical Methods, 2018, 10(2): 230-236.
6. Song H, Zhang Y, Wang S, et al. Label-free polygonal-plate fluorescent-hydrogel biosensor for ultrasensitive microRNA detection[J]. Sensors and Actuators B: Chemical, 2020, 306: 127554.
7. Bahari D, Babamiri B, Salimi A, et al. Graphdiyne/graphene quantum dots for development of FRET ratiometric fluorescent assay toward sensitive detection of miRNA in human serum and bioimaging of living cancer cells[J]. Journal of Luminescence, 2021, 239: 118371.
8. Celik Cogal G, Das P K, Yurdabak Karaca G, et al. Fluorescence Detection of miRNA-21 Using Au/Pt Bimetallic Tubular Micromotors Driven by Chemical and Surface Acoustic Wave Forces[J]. ACS Applied Bio Materials, 2021, 4(11): 7932-7941.
9. Zhang G, Zhang L, Tong J, et al. CRISPR-Cas12a enhanced rolling circle amplification method for ultrasensitive miRNA detection[J]. Microchemical Journal, 2020, 158: 105239.
10. Jiang W, Chen Z, Lu J, et al. Ultrasensitive visual detection of miRNA-143 using a CRISPR/Cas12a-based platform coupled with hyperbranched rolling circle amplification[J]. Talanta, 2023, 251: 123784.
11. Wang G, Tian W, Liu X, et al. New CRISPR-derived microRNA sensing mechanism based on Cas12a self-powered and rolling circle transcription-unleashed real-time crRNA recruiting[J]. Analytical chemistry, 2020, 92(9): 6702-6708.
